# Research for Policy (R4P): development of a reflection tool for researchers to improve knowledge utilization

**DOI:** 10.1186/s13012-016-0496-1

**Published:** 2016-09-30

**Authors:** Ingrid Hegger, Lisanne K. Marks, Susan W.J. Janssen, Albertine J. Schuit, Jolanda F.M. Keijsers, Hans A.M. van Oers

**Affiliations:** 1 National Institute for Public Health and the Environment (RIVM), PO Box 1, Bilthoven, BA 3720 The Netherlands; 2 Department of Health Sciences and the EMGO Institute for Health and Care Research, VU University, De Boelelaan 1085, Amsterdam, 1081 HV The Netherlands; 3 Tranzo Scientific Center for Care and Welfare, Tilburg University, PO Box 90153, Tilburg, 5000 TL The Netherlands; 4 Netherlands’ Organization for Applied Scientific Research TNO, PO Box 3005, 2301 DA Leiden, The Netherlands

**Keywords:** Research, Policy-making, Knowledge utilization, Reflection tool, Contribution mapping, Alignment areas

## Abstract

**Background:**

To improve knowledge utilization in policymaking, alignment between researchers and policymakers during knowledge production is essential, but difficult to maintain. In three previously reported case studies, we extensively evaluated complex research projects commissioned by policymakers to investigate how alignment is achieved in a research process and to discover ways to enhance knowledge contributions to health policy. In the present study, we investigated how the findings of these three research projects could be integrated into a practical tool for researchers to enhance their contribution to evidence-based policy.

**Methods:**

A cross-case analysis was conducted to integrate the findings of the evaluation of the three research projects and to identify important alignment areas in these projects. By means of an iterative process, we prepared a tool that includes reflection questions for researchers. The “Research for Policy” tool was tested with input from the project managers of three new research projects. Based on the findings, the final version of the Research for Policy tool was prepared.

**Results:**

By cross-case analysis of the three case studies, the following important alignment areas were identified: the *goal*, *quality*, *relevance*, *timing*, and *presentation* of research, the *tasks and authorities* of actors, the *consultative structure and vertical alignment* within organizations, and the *organizational environment*.

The project managers regarded the Research for Policy tool as a useful checklist for addressing the important alignment areas in a research project. Based on their feedback, the illustrative examples from the case studies were added to the reflection questions. The project managers suggested making the tool accessible not only to researchers but also to policymakers. The format of the Research for Policy tool was further adjusted to users’ needs by adding clickable links.

**Conclusions:**

Alignment between research and policymaking requires continuous efforts and a clear understanding of process issues in the research project. The Research for Policy tool offers practical alignment guidance and facilitates reflection on process issues, which supports researchers in aligning with policymakers and in acting in a context-sensitive way.

**Electronic supplementary material:**

The online version of this article (doi:10.1186/s13012-016-0496-1) contains supplementary material, which is available to authorized users.

## Background

Policy-making is a complex process, and the use of scientific knowledge in policymaking, also referred to as knowledge utilization, is not self-evident [1–3]. Not surprisingly, researchers want to know why the scientific knowledge uptake into policymaking is so difficult. For over 40 years, extensive research has been conducted on knowledge utilization, which resulted into several generations of theoretical models explaining barriers and facilitators [4–7]. Oliver et al. have pointed out that despite extensive research on knowledge utilization and many efforts to enhance scientific knowledge uptake in policy, barriers have been persistently identified in the literature, whereas only “little empirical data analyzing the processes and evidence use in policy is available” [8]. They argue that researchers should focus on understanding policymaking processes, the types of evidence used by policymakers, and the relationship between research and policymaking.

The first step for scientists is to recognize the characteristics of politics, policy, and policymaking. As Pielke describes, politics is the “process of bargaining, negotiation, and compromise” to determine how resources are allocated and under what conditions, whereas “policy” indicates “the commitment of a group to a particular course of action” aiming at a desired outcome for solving a specific problem [1]. Policy-making is thus the process to recognize the problem and to formulate, implement, and evaluate the particular course of action needed to solve the problem. To understand which scientific knowledge may help policymakers and contribute to policymaking and how this can be achieved, scientists have to acknowledge that policymaking is a dynamic, complex process in which different sources of knowledge and information are used and where the values and interests of various stakeholders have to be taken into account [7]. The components of the process, i.e., agenda setting of a problem, followed by formulation, implementation, and evaluation of policy, are often represented as successive stages in a policy cycle. However, policymaking is shown to be less linear and more iterative in practice [9–11]. In this complex process, scientific knowledge may have an indirect, not clearly discernible role: besides instrumental use in a direct and specific way to solve a particular problem, it may also have a conceptual function as a source of ideas or a symbolic/agenda-setting function when it is used to take an advocacy position [12–15]. Because of the dynamics of the policymaking process and the different purposes for which scientific evidence is used, it is generally recognized that formal and informal interactions between researchers and policymakers play a key role in the use of knowledge in policymaking [16]. The constructivist perspective on knowledge utilization even goes a step further. In this perspective, science is a social process during which scientific evidence is co-created by researchers and other involved actors, including policymakers [17]. As Kok and Schuit argue, this constructivist model is helpful in blurring the boundaries between research and policymaking and discovering ways to align research with policymaking in order to enhance knowledge contributions to policymaking [17].

Policy and policymaking may relate to various decision-making levels (national, local, governmental, or private). In this paper, the focus is on research for governmental policymaking by officials of a ministry or other governmental organizations at a national level. For scientists working in this context, the different perspectives on knowledge and the insights on knowledge utilization are particularly relevant. An example of a knowledge institute working for governmental organizations at national level is the National Institute for Public Health and the Environment (RIVM), the national public health institute in the Netherlands. RIVM conducts research and integrates knowledge in the field of public health, health care, safety, and environmental protection for governmental commissioning organizations such as the Ministry of Health, Welfare and Sport and the Healthcare Inspectorate [18]. RIVM and its clients have arranged formalized procedures to ensure agreement on research proposals, knowledge products, and timelines for commissioned projects. The knowledge products are important for making RIVM knowledge accessible to the target audience. They may take a variety of forms, such as reports, scientific papers, fact sheets, websites, or databases. In the RIVM annual commissioning cycle, RIVM researchers, specifically the project managers, consult the policymakers at the commissioning organization. These policymakers are their counterparts for a particular research commission and discuss the articulation of the knowledge question and formulation of the project plan. They have the task to arrange regular meetings with the commissioning client’s account manager and to gear their knowledge products to the commissioning client’s needs. RIVM management regularly monitors the progress of research projects and the timely delivery of products.

Despite these efforts to interact and align with the commissioning organizations, RIVM researchers have found that the contributions of their knowledge products to policymaking are not as significant as they expected. At the same time, commissioning organizations still indicate that they would like to be provided with knowledge products that are optimally aligned to their specific needs.

To investigate how alignment is reached and to discover ways of enhancing the contributions of RIVM knowledge to policymaking, we conducted a research project with two stages. In the first stage, we carried out three case studies, for which we used Contribution Mapping developed by Kok and Schuit [17, 19]. The case studies have been reported in three published scientific articles [20–22]. In the second stage of our research project, we translated the findings of the case studies into practical guidance by developing a reflection tool to support researchers in their alignment efforts. This second stage of the research is the subject matter of this paper. To provide background, we first summarize the findings of the first stage of our research project. We then continue with reporting the conduct and findings of the second stage.

### Summary stage 1: three case studies

**Fig. 1:**
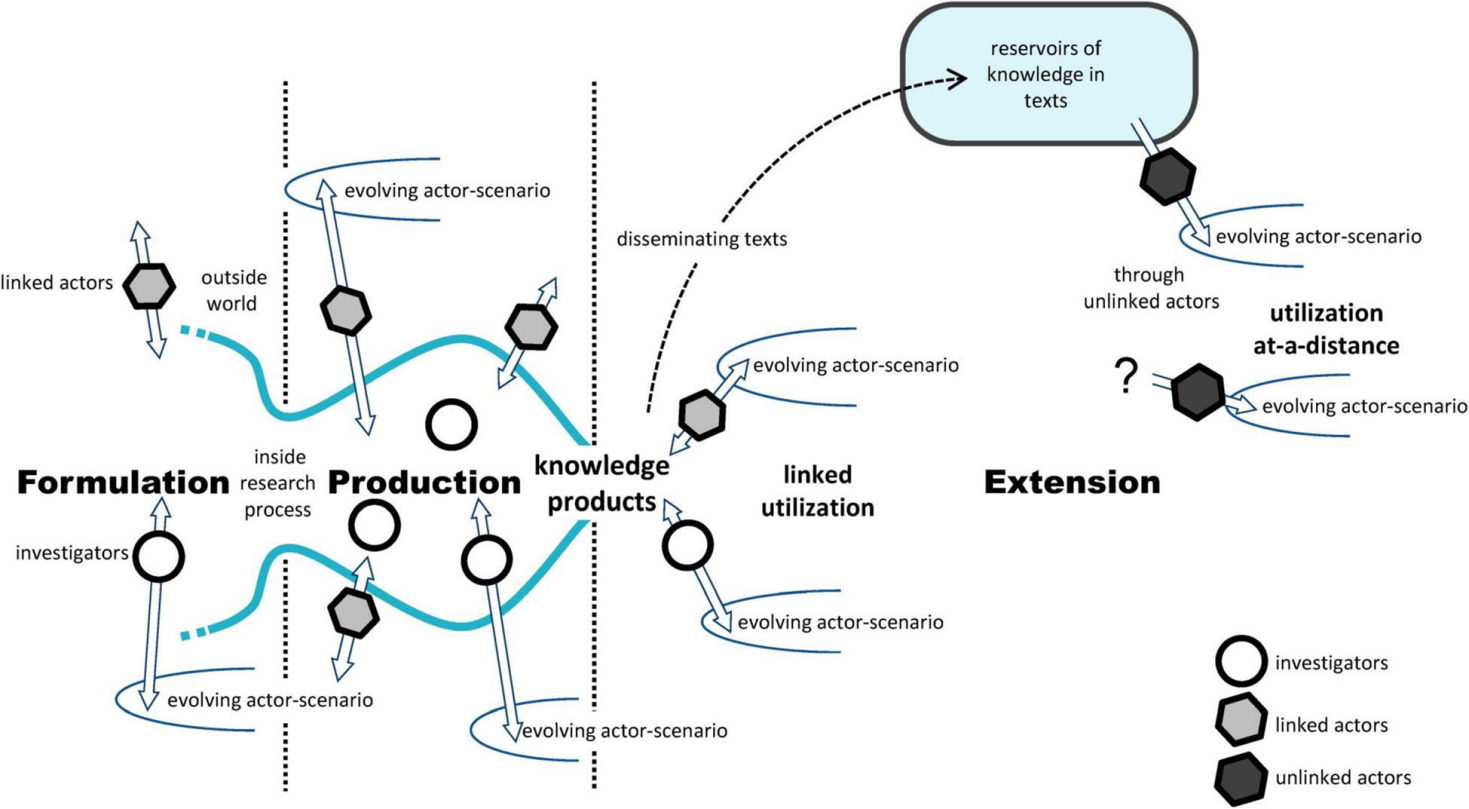
Kok and Schuit’s three-phase model

In the first stage of the research (2011–2013), we extensively evaluated three multi-annual RIVM research projects: “Development of Risk Model,” “Development of Dutch Health Care Performance Report 2010,” and “Development of Dutch Public Health Status and Forecasts Report 2010”. For each project, we analyzed the process in detail using the Contribution Mapping approach [17]. Based on a constructivist perspective, Contribution Mapping developed by Kok and Schuit is a method to evaluate the utilization and impact of knowledge generated by research projects in health policymaking. The method enhances the understanding of research processes and alignment between researchers and health policymakers. It is based on a three-phase model of the research process; a *Formulation Phase* to define the research question and plan, a *Production Phase* to conduct research, and an *Extension Phase* to disseminate the research results (Fig. 1).

Contribution Mapping conceptualizes the utilization and impact of knowledge through so-called *contributions to action*: “Contributions are activities that enable the conversion of knowledge into an element in decisions and implementation, a part of practices or a component in innovation” [17]. We consider the concept *contribution* valuable since it reflects that knowledge conversion into policymaking can take many, even very subtle, shapes and forms, already during the research process. The more comprehensive concept *knowledge utilization* may suggest that it is about the complete uptake of certain knowledge in policymaking, which may disguise partial, but meaningful use. To contribute to the work of actors, i.e., the persons or organizations involved, knowledge has to be included in the so-called *actor scenarios*. These are (virtual) scripts implicitly or explicitly formulated by the actors representing their view of the future and their pursuit. *Alignment* is an important concept in Contribution Mapping and means that research and policymaking are attuned and this is reached by reciprocal interaction instead of a one-way interaction from research to policymaking. To enhance the contributions of knowledge, alignment with actors and their actor scenarios is necessary. Specific actions can be taken for this purpose, so-called *alignment efforts*, defined by Kok and Schuit as “anticipatory efforts that aim to enhance contributions” [17]. Both researchers and policymakers can undertake alignment efforts. Due to some push and pull in this process, both positions may move resulting in knowledge and knowledge products (better) attuned to the needs of policymakers and researchers.

In each case study, we described the alignment efforts during the research process and the contributions that the project made to the policymaking process [20–22]. An outline of the three case studies is provided in Table 1 below. For further details, we refer to the research articles on the case studies [20–22].

**Table 1:** Outline of case studies

“Development of Risk Model” case study [20]
This case study focused on the development of a risk-based approach for clinical trial inspections in the Netherlands and had to deliver risk models to enable ranking and stratified selection of clinical trials for inspection by the Dutch Health Care Inspectorate. These models had to contribute to the Inspectorate’s objective of using scientific knowledge for evidence-based supervision. We found that RIVM and the Inspectorate had divergent views on their collaboration and the ownership of the knowledge product, which resulted in different expectations. Researchers and commissioning inspectors were not aware of these different perceptions. We identified six relevant categories of both horizontal alignment efforts (between investigators and key users) and vertical alignment efforts (within RIVM and the Inspectorate organization) that affected the contributions to the Inspectorate’s work. Relevant alignment efforts became manifest at three levels: the first level directly concerned the project, the second level concerned the organizational environment, and the third level concerned the formal and historical relationship between the organizations.
Case study on Dutch Health Care Performance Report [22]
The second case study concerned the Dutch Health Care Performance Report (DHCPR). The DHCPR is published by RIVM and commissioned by the Dutch Ministry of Health, Welfare and Sport in 2006, 2008, 2010, and 2014 [27]. Based on a scientific framework, the DHCPR monitors health care performance in the Netherlands by using indicators for quality, accessibility, and affordability. The aim of the report is to contribute to “strategic policymaking”. We identified six areas where alignment is specifically relevant for enhancing the contributions of future DHCPR editions: well-balanced information for different ministerial directorates, backstage work, double-role actors, reports published by other knowledge institutes, data collection and generation, and presentation formats.
Case study on Public Health Status and Forecasts Report [21]
The third case study concerned the Dutch Public Health Status and Forecasts Report (PHSF), which integrates research data and identifies future trends in public health in the Netherlands [28]. The PHSF has a recognized function in connecting the science and policy domains because it is embedded in the national health policy cycle by law. The PHSF provides the policy themes for the National Health Memorandum (NHM), which is published every 4 years by the Minister of Health, Welfare and Sport. The PHSF2010 process included activities aimed at alignment between researchers and policymakers, such as informal meetings. However, we identified three issues that are easily overlooked in knowledge production, but provide suggestions for enhancing contributions: awareness of divergent, continuously changing actor scenarios; vertical alignment within the organizations involved; and careful timing of draft products to create early adopters.

### Purpose of this article

In the first stage of our research project, we used the Contribution Mapping method in three case studies to gain insights into areas where specific alignment efforts can be helpful to enhance contributions of RIVM knowledge and knowledge products.

In the second stage, we translated the findings of the first stage by developing a tool to support researchers in their everyday work, since the insights from the case studies did not directly provide guidelines for improving alignment within future projects. The case studies had shown that alignment between researchers and policymakers is more difficult to maintain in practice than expected. Moreover, the need for alignment efforts continuously changes, depending on the research project and its current phase. We concluded that the key to enhancing alignment efforts and therefore the contributions of research is the researchers’ awareness of key alignment areas, combined with regular, systematic reflection. This involves taking sufficient time to consider seriously the current situation of the project, taking into account the alignment areas [20, 22].

We acknowledged that it would not be helpful to offer generic alignment efforts, since every research project is different. Alignment efforts are difficult to predefine in a tool since they depend on the specific research process (both the knowledge question and the project’s context, i.e., the environment of the project that influences the process and outcome of the project) at a certain point in time, making it impossible to suggest beforehand any detailed (inter)actions applicable to each research project. Furthermore, we concluded that the continuously changing context of research projects requires regular reflection on the research process to identify the need for specific alignment efforts. We found that researchers find it difficult to analyze regularly the process issues of their project, often due to unawareness of important alignment issues, time constraints, and their preference for scientific issues.

**Fig. 2:**
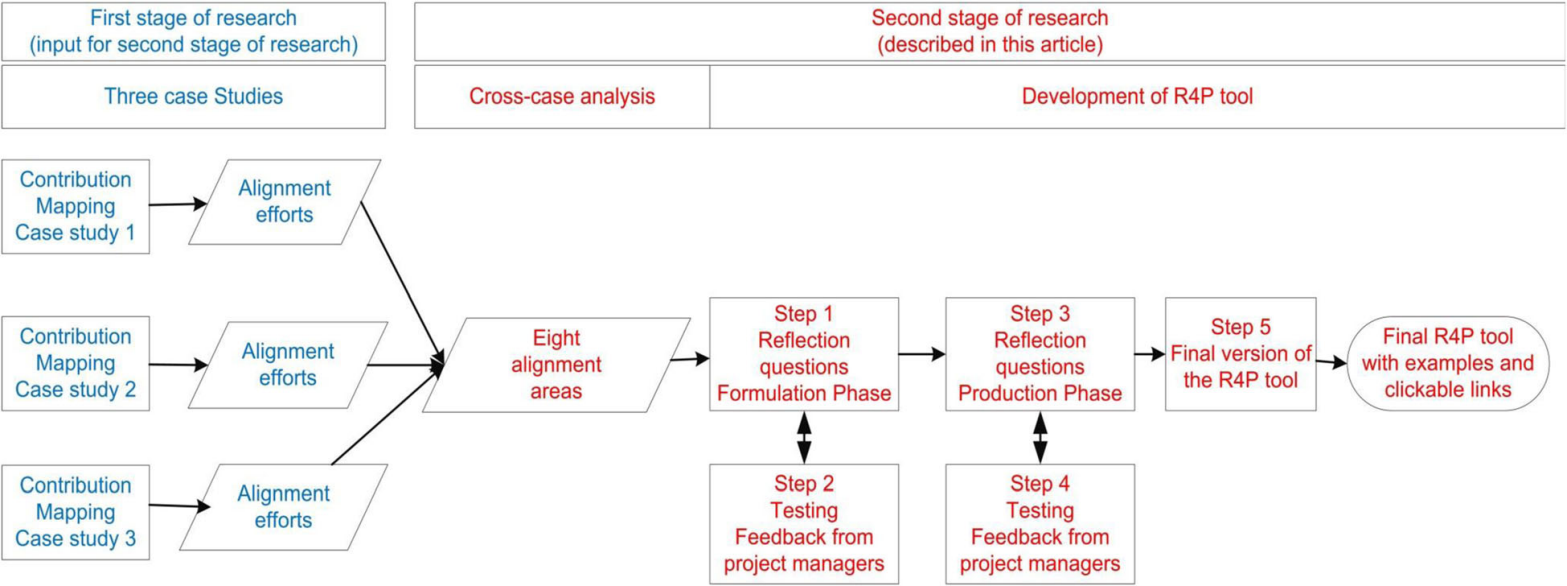
Process of the development of the R4P tool

In this article, we report the results of the second stage of our research project. Firstly, we describe how we integrated the findings of the three case studies of the first stage by conducting a cross-case analysis. Secondly, we describe how we developed the Research for Policy (R4P) reflection tool to support researchers in creating alignment with policymakers and preparing aligned knowledge products. For the formulation of the specifications of the R4P tool, we took into account the insights acquired in the first stage. The process for the development of the R4P tool is outlined in Fig. 2.

## Part A: Cross-case analyses

### Methods

We systematically analyzed the alignment areas of the three cases as identified in the first stage of our research project by a case-oriented approach [23]. In line with the Contribution Mapping approach, we described the alignment areas in a table for comparison and drew up a comprehensive list of important alignment areas [17, 19].

### Results

The cross-case analysis of the three case studies revealed eight areas where deliberate alignment efforts could specifically improve alignment between RIVM and the commissioning Ministry of Health, Welfare and Sport (see Table 2). Based on the barriers indicated by de Goede et al. and general alignment efforts formulated by Kok and Schuit, the alignment areas “goal,” “quality,” “consultative structure,” “relevance and timing,” and “presentation” were anticipated findings [16, 17]. The alignment areas “tasks and authority,” “vertical alignment”, and “organizational environment” were additional areas.

**Table 2:** Consolidated list of areas for alignment

Area for alignment	Topics
Goal	The formulation of the knowledge question; exploration of its origin, the “question behind the question,” and the underlying need for the knowledge products
Tasks and authority	The input of all involved actors (both researchers and policymakers); their responsibilities, knowledge and data exchange by actors during the process, and the final authority over the knowledge products
Quality	The research method; conceptual framework and data used in the research project
Consultative structure	The consultative structure of the project; the sharing of relevant information and the relationships between actors; double-role actors
Vertical alignment	Interaction within the organization conducting research and within the commissioning organization; interaction between hierarchical levels and the embedding of the project in the organizations
Organizational environment	The environment of the research project; awareness of relevant conditions external to the research project influencing the relationship between investigators and linked actors; incidents, media events, relationships with other organizations, changing priorities, and changing actors
Relevance and timing	The formulation and wording of the research results and timing of the delivery and presentation of the knowledge products
Presentation	The design and structure of knowledge products and the tools for the extension strategy

The alignment areas represent the topics where researchers should reflect on to achieve sufficient alignment before and during the research project. In this way, they will be able to determine whether they should put more effort into alignment.

- Alignment area *Goal* represents the need for full clarity regarding the expectations about and the purpose of the knowledge product.
- Alignment area *Tasks and Authority* indicates that a project manager should define the tasks and responsibilities within his/her research team and should also assure a clear agreement on the rights and responsibilities of both the commissioning organization and the research institute within the project to avoid debate at a later stage.
- Alignment area *Quality* implies that the scientific models, concepts and definitions used during the research can have a significant bearing on the findings and need agreement already at the start. This is to avoid dismissal of an unfavorable research result by arguing that the approach was flawed.
- Alignment area *Consultative Structure* focuses on the need to ensure both capacity and time to allow for adequate interaction during the research process. There is also attention for planning exchange of knowledge with intended users.
- Alignment area *Vertical Alignment* stresses the importance of vertical embedding of the product within the organization for its legitimacy towards the commissioner and the resources and capacity required. It is essential to refer any problems, difficulties, or obstacles, which cannot be resolved at the research level to a higher hierarchical level. Conversely, the higher hierarchical level should inform researchers on specific issues that may influence there project.
- Alignment area *Organizational Environment* is about identification of the characteristics of the different involved organizations. The institutional work culture of the commissioning organization may influence the process and the dynamics of its context are important to understand the commissioner’s motive. Furthermore, developments in the own organization should be well known and understood.
- Alignment area *Relevance and Timing* points out that the commissioner’s requirements with regard to the purpose, form, and timing of the product may change over time, even after approval of the original project proposal. Any changes to the process itself may then be necessary. Furthermore, awareness about any political sensitivity with respect to wordings and formulation of research results is important.
- Alignment area *Presentation* reflects the need to agree timely on the form and presentation of the knowledge products and their dissemination.

## Part B: Development of the R4P Tool

### Methods

We developed a reflection tool for researchers called R4P with the purpose to support researchers in reflecting on their project to identify the need for alignment efforts during the research process. Based on the findings in the first stage as described in the “Background” section, we formulated the following specifications:

- The R4P tool should support users in engaging in systematic reflection on the research process.
- The R4P tool should take into account the alignment areas identified in the case studies.
- The R4P tool should be generally and easily usable at project team level.

In order to comply with the specifications, we decided to include open-ended questions that would promote awareness and critical reflection on the process aspects of the project, while taking into account the alignment pitfalls that we identified in the case studies by contribution mapping. In this way, we intend to increase researchers’ sensitivity to the context of their project and to the policymaking process. As they become more context-sensitive, researchers will be more capable of recognizing and acting on the importance of alignment with the commissioning and other organizations, in order to enhance the impact of their work. At the same time, they will be more capable of acknowledging the different roles and responsibilities of researchers and policymakers, and taking into account the need to stay independent.

### Step 1: Preparing the first part of the R4P tool

We first prepared the part of the R4P tool, which focuses on the Formulation Phase of a project. In iterative, open sessions, our research team, consisting of the two acting researchers and their supervisors (two senior researchers and two university professors), discussed the formulation and categorization of the open-ended reflection questions and the accompanying explanation for each question until consensus was reached. They were concisely formulated and we added a brief explanation to each question to provide some background concerning the question (Additional file 2: Draft R4P tool, part Formulation Phase).

### Step 2: Testing the R4P tool during the Formulation Phase

We asked three experienced project managers who work on policy-orientated research projects in the field of public health and health care to test the Formulation Phase questions of the R4P tool for usability in their projects during the upcoming new project cycle (2013/2014). In August 2013, each project manager received a verbal explanation of the R4P tool. They were asked to use the tool in the Formulation Phase of new projects for the year 2014. After 3 months, they were interviewed face to face and asked to provide feedback on the tool (Additional file 1: Topic list). Each semi-structured interview took approximately 1 h and was recorded and analyzed by mapping.

### Step 3: Developing an extension of the R4P tool for the Production Phase

For use in the Production Phase, we developed an extension of the R4P tool in a similar process as the development of the Formulation Phase part. Based on the project managers’ feedback on the part for the Formulation Phase, we added examples to the Production Phase questions. The examples illustrate the rationale of each question and concretize the rather general wording of the questions (Additional file 2: Draft R4P tool, part Production Phase).

### Step 4: Testing the extension of the R4P tool during the Production Phase

Again, we asked the same three experienced project managers to use the R4P tool in the production phase of their project during the year 2014. They were interviewed face to face and asked to provide feedback on the tool (see Additional file 1: Topic list). Each semi-structured interview took approximately 1 h and was recorded and analyzed by mapping.

### Step 5: Preparing the final version of the R4P tool for all phases

To complete the R4P tool, we integrated the Formulation Phase and Production Phase questions and added examples from our case studies to the questions of the Formulation Phase. Based on the project managers’ feedback, we decided to organize the questions according to content rather than by project phase. We classified the questions into four categories: organizational environment of the project (I), goal of the project (II), interaction during the project (III), and outcome of the project (IV).

In the final version of the R4P tool, we provided an overview to indicate in which phase of the research project each question could be relevant, and we included clickable links to the questions. The questions themselves also contain clickable links to explanatory notes to the questions, as well as corresponding examples (Additional file 3: Final R4P tool).

### ResultsStep 2: Testing the R4P tool during the Formulation Phase

In step 2, we tested the part for the Formulation Phase, which included ten questions in five categories (Additional file 2: Draft R4P tool, part Formulation Phase). When providing their feedback on this part of the draft R4P tool, the project managers regarded the topics raised in the open-ended questions as the key topics to address during the research process. However, they indicated that they experienced the questions as rather abstract, which made it more difficult to link them to their daily practice. Therefore, we added examples to the question for the Production Phase.

### Step 4: Testing the extension to the R4P tool during the Production Phase

The project managers considered the examples provided in the Production Phase questions to be very helpful in clarifying the questions and in finding an approach for alignment in comparable situations. They pointed out that they experienced some overlap between the Formulation Phase and Production Phase questions and stated that they regarded the tool as already suitable for (anticipating on) the extension phase. They were in favor of a single integrated R4P tool with questions for all phases. After all, they considered the R4P tool a useful checklist for addressing the important process topics of a research project. They indicated that the R4P tool should be readily accessible not only to health-systems researchers but also to researchers in other expertise domains and to RIVM are commissioning clients, such as policymakers working at the Ministry of Health, Welfare and Sport. The project managers suggested using the R4P tool for education of project managers as part of the regular project management courses. They also suggested making the tool more user-friendly, for example, by making it available online and adding clickable links.

### Step 5: Final version of the R4P tool for all phases

Based on the feedback of the project managers, we decided to merge the two parts of the R4P tool into one list of questions for all phases. The final version of the R4P tool contains 23 reflection questions and is presented in an additional file (Additional file 3: Final R4P tool). The list of questions contains clickable links to explanatory notes to the questions and corresponding examples. The examples have been derived from our case studies and reflect real-life situations recognizable for RIVM researchers. Most questions are relevant for both the formulation phase (21 of 23) and the production phase (22 of 23), whereas ten questions are relevant for the extension phase. To indicate in which phase of the research project each question can be relevant, the first page provides an overview including clickable links to the questions.

## Discussion

The Contribution Mapping approach in our case studies provided us with useful insights into important areas for alignment between researchers and policymakers in the field of public health and health care. We integrated the alignment areas into one list by a cross-case analysis. In line with the work of de Goede and of Kok and Schuit, the anticipated alignment areas “goal,” “quality,” “consultative structure,” “relevance and timing,” and “presentation” focus on the optimal interaction between researchers and policymakers [16, 17]. However, the identified additional areas “tasks and authority,” “vertical alignment,” and “organizational environment” are more connected to the context of the research project. We found that these context topics strongly influence optimal interaction and alignment with policymakers. Researchers were not always aware of the context in relation to their project and of its influence on their own interaction and alignment with policymakers. Therefore, it is important for researchers to comprehend context issues in relation to their project and to act upon them in aligning with policymakers.

We argue that the R4P tool can be useful for other public health and health systems research projects, since it focuses on process issues that are relevant to most research projects. The case studies enabled us to translate the alignment areas into reflection questions and illustrative examples that can be used in daily practice. Furthermore, we identified several important topics that relate to improving the contributions of scientific knowledge to policymaking and that offer ways to facilitate alignment efforts and thus enhance contributions to evidence-based policymaking. These findings can be of interest for researchers conducting research in commission of governmental organizations.

### All phases of the research process require alignment efforts

For an individual researcher, alignment with policymakers is the most practical way to influence the contributions of their research and knowledge [24]. Schut et al. also concluded that researchers have to address the challenges of the complex dynamics of policy-oriented research through reflection and context sensitivity [25]. Alignment requires awareness of emerging issues and continuous efforts at various levels. Both the case studies and the exploratory pilot project showed that personal interactions between individuals—both in the organization conducting the research and in the commissioning organization—are crucial for alignment. In our case studies, personal interactions between researchers and policymakers occurred mainly during the production phase of a research process, being the lengthiest phase. However, we want to point out that informal interactions during the formulation phase and the extension phase are equally important. In the formulation phase, interaction can prevent an unarticulated knowledge question, a request for research being so imprecisely formulated that it does not represent the exact knowledge need and may have a negative impact on the outcome of the project beforehand. At the end of the production phase, knowledge is mostly presented in a knowledge product, such as reports, scientific papers, websites, or databases. In the extension phase, interaction may ensure that the level of interest in the knowledge products does not decrease quickly.

We acknowledged that project managers have to align on topics relevant for the Extension Phase already during the Formulation and Production Phase. Although the Three-Phase-Model proved to be helpful in the analysis of the case studies, we departed from the separation into three phases for the R4P tool. One list of reflection questions turned out to be more convenient than a list for each separate phase due to the overlap in topics.

### Alignment has to be organized

To facilitate better alignment in all phases, knowledge institutes should pay explicit attention to and recognize the everyday process issues associated with knowledge production. For knowledge institutes, it can be very useful to analyze their organizational routines and reserve budget for developing awareness of context issues and interactions with policymakers. Individual researchers will be encouraged to pay attention to the process issues associated with research if they are rewarded for doing so, for example, when sufficient time and appreciation is devoted not only to the scientific merits of their work [26]. For commissioning organizations, it is just as important to take the need for organizing alignment into account.

### Vertical alignment is crucial

Our experiences in our research suggest that many researchers prefer to pay attention to scientific issues rather than spending time to alignment. Thus, the urgency of devoting attention to alignment must be made clear to researchers, since a single, well-aligned knowledge product that really offers a contribution will often be preferable to a large number of knowledge products that are of little practical use. However, this aim cannot solely be achieved at research level, but requires efforts and collaboration at all hierarchical levels of both organizations (i.e., the knowledge institute and the Ministry) during the entire research process. It is crucial that all parties involved are aware of their role in the research process and assume their responsibilities while taking into account those of the other organization. Although alignment is essential to enhance contributions to policymaking, knowledge institutes must simultaneously balance adequate alignment with policymakers with sufficient distance from the policymaking domain if they wish to maintain their independence. Internal vertical alignment between researchers, project managers, and line management of the knowledge institute is essential to optimize both alignment and independence and to strike the right balance.

### Usability of the R4P tool

During our project, we found that theoretical considerations on alignment and knowledge contributions have little appeal to health systems researchers. Therefore, the understanding of important alignment areas and the topics they represent had to be “translated” in order to be useful to researchers in their everyday work. In the R4P tool, the combination of reflection questions based on research findings and illustrative examples from the case studies with situations familiar to the researchers particularly offered added value.

Although our reflection tool is based on translation of alignment areas derived from case studies in our own institute, we argue that the tool could also provide a basis for reflection in other research projects, both for researchers and policymakers, since the topics covered are process-related and the tool can be used in a flexible way. The present tool includes 23 questions that cover all important alignment areas. It is up to users to decide whether they are relevant to their particular project at a given point in time and whether the answers to the questions have to result in an alignment effort.

## Conclusions

Aligning to the needs of policymakers offers researchers the opportunity to take influence into their own hands in an ever-changing context, such as the policy priorities of the Ministry, the influence of political reality on the relevance of their knowledge products, and the organization of their own research institute. We found that reaching alignment is not easy at all and depends on many aspects. In our study, we first identified the most important aspects for researchers in government-commissioned research. By cross-analysis of the case studies, we could identify eight key alignment areas: the goal, quality, relevance, timing, and presentation of research (findings), the tasks and authorities of actors, the consultative structure and vertical alignment within organizations, and the organizational environment. The R4P tool is based on these areas. We intended to develop an instrument that supports researchers in undertaking alignment efforts to enhance the contributions of their work to policymaking. Researchers recognized the questions—which were illustrated with examples from case studies—as relevant and to the point. The R4P tool can be deployed in any health systems research project to reveal the topics that are most important in the project. Initial experiences in using the R4P tool show that it offers useful alignment guidance to researchers and facilitates reflection on process issues, which will help researchers to adopt a more context-sensitive approach in their work. By regular reflection, researchers will be better able to decide what to do or not.

Since the questions in the tool are intended for inspiring reflection, it is not necessary to answer completely all questions at the same time. It is up to the users which topic they want to reflect on depending on the phase and characteristics of a specific research project. By thinking about the answer to the questions, researchers gain insight into the ongoing process and become aware of what action is needed and how to anticipate on the policymaking reality. The tool can be used as a checklist by individuals and as basis for open discussion in project teams. The R4P tool may also provide a basis for dialogue between researchers and policymakers, turning it into a shared tool for alignment. For the use of the R4P tool as shared guidance in the dialogue between researchers and policymakers, the next step will be to assess its applicability for policymakers and any need for adaption. Finally, the ultimate step will be to investigate the influence of using the R4P tool on actual knowledge contributions to policymaking.

## Additional files

Additional file 1: Topic list. (DOC 28 kb)

Additional file 2: Draft R4P tool. (DOCX 30 kb)

Additional file 3: Final R4P tool. (PDF 358 kb)

## Abbreviations

R4P tool: Research for Policy tool
RIVM: National Institute of Public Health in the Netherlands (Rijksinstituut voor Volkgezondheid en Milieu)
