# Supplementary material for: Research for Policy (R4P): development of a reflection tool for researchers to improve knowledge utilization
Source: Implement Sci. 2016 Sep 30;11:133. doi: 10.1186/s13012-016-0496-1 (PMC5045649; doi:10.1186/s13012-016-0496-1)
Supplement: Supplementary file 1 — Topic list. (DOC 28 kb) [file 13012_2016_496_MOESM1_ESM.doc]

**Additional File 1**

**Topic list for interviewing researchers on R4P tool in step 2 (after Formulation Phase) and step 4 (after Production Phase)**

***Introduction***Short introduction of the research project Improving Knowledge Utilization, the R4P tool and the pilot

Goal and structure of the interview.

**Content R4P tool** **(general)**

First impression of the content;

General characteristic of the questions according to interviewee (e.g. difficult, helpful, reflective, etc);

In relation to your own project: most / least suitable questions (reason); lacking questions (reason); irrelevant questions (reason);

General applicability questions (other projects)

**Use and contributions of the R4P tool** **(specific)**

Use: when; how

Contributions: discussed with someone? ; usefulness for own project; target group

**View of interviewee on project management**

Most important issues (reason); difficult issues (reason)

Acquaintance with commissing organisation: culture; tasks and activities; interaction with respect to knowledge question and intended use

**Points for Improvement of the R4P tool**

Contributions to projects: how; conditions; other presentation forms
Comparison R4P tool in step 2 and step 4: preference; opinion on examples

Dissemination R4P tool: suggestions
