# Supplementary material for: Research for Policy (R4P): development of a reflection tool for researchers to improve knowledge utilization
Source: Implement Sci. 2016 Sep 30;11:133. doi: 10.1186/s13012-016-0496-1 (PMC5045649; doi:10.1186/s13012-016-0496-1)
Supplement: Supplementary file 2 — Draft R4P tool. (DOCX 30 kb) [file 13012_2016_496_MOESM2_ESM.docx]

**Additional File 2: Draft R4P tool**

**Formulation Phase**

**Alignment Area Stake holders: organizational environment; consultative structures; tasks & authorities**

1a) What organizations will be involved in the project and in what capacity? What is the position of these organizations within the health domain of the project, and what are the practical implications of its position?

*Explanation 1a) It is important to identify: What are the characteristics of the different organizations in question? What is their position (in relation to your organization’s network), what authority or influence do they possess, and how can this be exploited in the interests of the project?*

1b) What are the specific characteristics of the researchers on your project team? How are tasks and responsibilities divided between them, and how is the work coordinated?

*Explanation 1b) It is important to consider: Are all necessary competences and expertise represented within the team? Who are the designated specialists (for communications, media, design, data collection, etc.)? Are all team members aware of the purpose and deadlines of the product?*

1c) What are the (current) characteristics of the commissioning organization and its relationship with your organization? How do you address these factors during the process?

*Explanation 1c) It is important to identify: What are the characteristics of the commissioning organization (e.g. Health Care Inspectorate, Ministry of Health, or other ministry), including its institutional work culture? Current contextual developments which involve or affect the commissioner should also be identified (e.g. reorganization, political developments, media attention). Attention should also be devoted to the commissioner’s role within the research process (e.g. as a member of a steering group or advisory committee) and the degree of ‘formal’ involvement.*

**Alignment Area Knowledge question: goal; relevance& timing**

2 a) How and when should the research product be used according to your organization and the commissioning organization? What is its purpose?

*Explanation 2a) You need full clarity regarding the expectations which the product is to meet. In the case of a research report for the Ministry of Health, for example, the parties should agree the point within the policy process at which it is to be used, and the nature of that use.*

2b) What is the underlying concern of the research question in relation to the problem that the commissioner wishes to solve?

*Explanation 2b) You need clarity with regard to whether the research question and its interpretation adequately address the commissioner’s need. Conversely, and depending on the type of product and the anticipated results, it may also be necessary to examine whether the commissioner will be able to implement an adequate response based on the research findings and whether adequate policy instruments are available to do so. You have to be aware that the commissioner can be confronted with queries or criticism on the research from the professional field, parliament, the media, etc.*

2 c) To what extent is it possible to refine the research question at a later stage of the research process to bring it more closely in line with the commissioner’s need?

*Explanation 2c) A ‘broad’ research question is not necessarily a bad thing since you will have greater discretion to define your own focus, which will emphasize the organization’s independent scientific position. You don’t have to specify all research requirements in advance if you create opportunities for further refinement and coordination during the research process (e.g. by means of scheduled consultation meetings).*

**Alignment Area Position project in internal organization: vertical alignment; quality; presentation**

3) With whom should you align within your own organization during the process? Whose commitment must be sought and how?

*Explanation 3) Vertical imbedding of the product within your organization is important for its legitimacy towards the commissioner, the resources and capacity required (determined in part by the desired quality and form of the product) and own future use or further research.*

**Alignment Area Position project in external organization: vertical alignment; presentation; quality; consultative structure**

4 a) With whom should you align *externally* during the process and whose commitment must be sought? How do you intend to do so?

*Explanation 4 a) In advance of the research process, you should identify the persons with whom alignment is required (also with a view to the intended recipient of the product). You should also determine how contact can best be established and maintained.*

4 b) What type and frequency of consultation with the commissioner will be most appropriate to the process and why is that?

*Explanation 4b) You should ensure that both capacity and the time schedule allow for adequate interaction and consultation. Agree the form that such consultation will take (e.g. face-to-face, via social media, email etc.) with all parties concerned.*

**Alignment Area Users’profile: vertical alignment; relevance & timing; presentation**

5) What types of user (at various levels within the commissioning organization) can be identified for the intended product, and how is their diversity to be taken into account in terms of content and process?

*Explanation 5) There may well be several types of user within the commissioning organization (e.g. different policy directorates, departments or hierarchical levels). Their diverse interests may demand differentiation in terms of the presentation of the product, both in form and timing.*

**Production Phase**

**Alignment Area Check project plan: Goal; relevance & timing; quality**

1a) Has the commissioning organization experienced any changes which may affect their knowledge need since the project proposal was produced? Does the research question formulated in the project proposal continue to address the current knowledge need? Is it necessary (and possible) to amend the project plan?

*Explanation* 1a) *The commissioner’s requirements with regard to the purpose or form of the end product may change over time, even after approval of the original project proposal. It is important to ascertain whether this is the case on a regular basis, and to consider whether any changes to the process itself are then necessary (and possible).*

*Example: A commissioner wishes to commission research examining the risks of a new technology used in hospitals, as a follow-up to an earlier study. A proposal is produced but before the project itself is implemented, a serious incident involving a different type of technology takes place. This causes significant media attention and the commissioner is subject to severe criticism. It is now far more important for the commissioner to gain further knowledge about this new technology than about that covered by the original proposal. In consultation with you, a new focus and a different approach are adopted.*

1b) Within what timeframe does the commissioner require the product and why? Did you agree on the project schedule? Are all phases, milestones and deliverables clearly defined?

*Explanation* 1b) *The exact moment of the product’s finilization and/or presentation can be important in terms of its relevance and use.*

*Example: the commissioning Ministry requires a research report to support an important policy document which is subject to a very strict deadline. Finilization after the deadline will make the product far less relevant to the commissioner and therefore, the project team agrees on the minimum part of the product to be delivered before the deadline.*

1c) Have the scientific models, concepts and definitions to be applied within the project been agreed by all parties?

*Explanation* 1c) *The scientific models, concepts and definitions used during the research can have a significant bearing on the findings. It is possible to dismiss an unfavourable research result by arguing that the approach was flawed. To ensure the acceptance and relevance of the product, it is prudent to create support for the scientific basis among all stakeholders, including the commissioning organization. It is also important to identify any (political) sensitivities about the models or definitions.*

*Example: You find that a certain type of medical care has various definitions in practice, with different interventions and equipment being grouped under the same general heading. For the purposes of your research, you opt to apply the definition followed by the majority of health care professionals and for which reliable data sources are available.*

*When the draft report is completed, the high cost calculation takes the commssioner unpleasantly by surprise. The commissioner argues that your organization did not apply the correct definition and objects to the conclusions being published in this form.*

**Alignment Area Context of the project: Organizational environment**

2a) In what (dynamic) context does the commissioner operate during the project, and what implications could this have for the project itself?

*Explanation* 2a) *It is essential to monitor the context in which the commissioner is operating to understand any unexpected course of action and to be able to discus this with a view to understanding the commissioner’s motives.*

*Example: At the commencement of your research project, the commissioner’s contact person asks you to be kept fully informed of the decisions and choices made by your research team. Two months later, it turns out to be impossible to contact her in time, because she does not answer the phone, responds to emails only after a lengthy delay and, according to her secretary, has no time to schedule a meeting. Meanwhile, there have been media reports of an urgent reorganization within the commssioning organization, prompted by the minister’s desire to show effective leadership following a number of incidents. This clarifies why the contact person is preoccupied with other matters. You align with the head of department and the account manager to decide how best to proceed.*

2b) In what dynamic context does your institute operate, and what implications could this have for your project and the alignment with the commissioner?

*Explanation* 2b) *External parties generally regard your research institute as a single, integrated organization. It is however important to devote attention to the activities and developments at the various levels within your institute and its individual departments in order to ensure uniformity, avoid overlap and address circumstances which may affect your institute as a whole. Matters to be considered include strategic plans, alignment between your institute and commissioning organizations at management level, new tasks and responsibilities for your organization, and other research projects or reports.*

*Example: Your institute publishes a report about the adverse effects of cycling in traffic, notably the inhalation of fine particulate matter from exhaust fumes. A week later, your institute publishes another report in which it advises local authorities to encourage people to cycle to work as a form of healthy exercise. The institute has unintentionally issued conflicting messages, causing confusion and attracting adverse media attention.*

**Alignment Area Interaction during the project: Tasks & authorities; Relevance &timing; Organizational environment.**

3a) Did you plan any interim knowledge exchange with the commissioner regarding the content of the research product?

*Explanation 3a) The intended effect of the research product can be increased or achieved somewhat sooner by scheduling discussions of the interim results (before completion of the project and its final report). It is important to discuss possible research outcomes with the commissioner at the earliest possible opportunity, in order to allow all parties to be adequately prepared for the implications of a particular finding.*

*Example: Your institute foresees that the research results will not be politically expedient. Provided there is timely discussion with the commissioner, the ministry can prepare an adequate response before the report itself is published. It must be remembered that such discussion relates solely to alignment and anticipation. It does not entail the commissioner’s ‘approval’ of the results. Your institute maintains its scientific independence and integrity by ensuring appropriate internal alignment between all hierarchical levels of the organization.*

3b) How do you achieve interim exchange of knowledge between the various users?

*Explanation 3b) The impact of the research product will be enhanced when there is interim discussion and an exchange of knowledge between the various end users during the course of the project. It is therefore useful for the project coordinator to know who alignst with those end users on behalf of the commissioner, and what activities he or she undertakes in order to do so. If the commisioner’s alignment efforts are not enough to ensure knowledge exchange with the end users, it may be necessary for the project team to take action, involving the account manager and his/her staff.*

*Example: A project has a steering group on which various directorates of the Ministry of Health are represented. The project group collates and processes the steering group’s comments on the draft versions of the final research report. Some directorates offer considerable feedback, others little or none at all. The evaluation reveals that the directorates which have provided least feedback also show least awareness of the report’s findings, yet are the most critical with regard to its practical value. A steering group member who shows considerable involvement and engagement in the process tends to engender greater satisfaction. The project team decides that any follow-up project will involve closer alignment with the commissioner’s contact person in order to determine the exact membership of the steering group and the degree of involvement its members are expected to show.*

**Alignment Area Planning of the project: Relevance &timing; Organizational environment**

4a) How do you monitor the production of (similar) knowledge products by other organizations, and the influence that such products may have on the commissioner and end users during the project?

*4a) Throughout the production phase, it will be useful to ascertain whether other research organizations are working on similar or related products. It is also important to determine the likely value of such products to the commissioner, and whether they will affect the (perceived) relevance of your product. This will enable you to take these influences into account in your project and in the alignment with the commissioner.*

*Example: Another research organization intends to present a report at a large event just days before you are due to publish a product relating to similar subject matter. This will inevitably distract attention from your product. Moreover, inside information suggests that the conclusions of the other report directly contradict your own findings. This could place the commissioner in a particularly difficult situation. You decide to contact both the commissioner and the higher hierarchical levels within your institute to align on the potential problems and the action required.*

4b) Do you have a timely discussion with the commissioner with regard to the form of the research product?

4b) *Much research culminates in the publication of a report. However, it is possible that a different form of product will be more useful to the commissioner. Examples of alternative products include a summary of a scientific publication to support a policy decision, a presentation, or an instrument such as a simulation model or a survey questionnaire. Knowledge presented during a round-table discussion is also a ‘product’.*

*Example: A commssioner faces a particularly complex policy issue and requests your institute to provide input for its internal deliberations. Following consultation with the commissioner, it is agreed to prepare and host a brainstorming session for the policy-makers concerned. Based on desk study of the existing literature, your team will also produce a background document and a presentation. On the outcomes of the brainstorming session, the commssioner is able to base further action and decisions, and also formulates terms of reference for a research project on the same topic to be conducted the following year.*

4c) Do you envisage to draft a plan for the presentation and dissemination of your research product, and has this been agreed well in advance with both the commissioner and relevant internal staff?

*4c) Working to a set plan for the presentation and dissemination of the research product will increase its outreach and hence the likelihood that the findings are acted upon in practice. It is useful to plan the relevant activities during the course of the project itself, since there may be neither time nor capacity once the product has been completed*

*Example: Despite some delays in a project, the deadline can still be met if staff works overtime. The research product is completed just before the Christmas holiday, much to the relief of the project team. When work resumes in January, the team is disbanded and its members assigned to new projects. They have no time left for extended presentation or dissemination of the former project’s results since these activities had not been planned and no budget is available. During the new commissioning cycle by the Ministry, the researchers realize that their previous research efforts did little to raise awareness of their findings. The policymakers only show scant interest in the research findings and the Ministry commissions only limited further research.*

**Alignment Area Vertical alignment**

5a) Did you explicitly agree on the rights and responsibilities of both the commissioner and your institute within the project?

*5a) General rules establish the rights and responsibilities of the parties involved in a project of your institute. However, it is also important to examine whether any specific agreements are required with regard to aspects such as the ownership of data, the procedures for providing feedback on draft versions of the product, and intellectual property rights.*

*Example: A commissioning organization wishes to have three of its staff review a draft report. However, one of the three is extremely slow to do so. Clear agreements have been made with regard to the review process and the action to be taken further to the reviewers’ comments (Your institute retains full editorial control and will decide whether to make any suggested changes). In addition a time limit been set, i.e. the annotated version must be returned within two working weeks. The research coordinator now presents the commissioner’s contact person with two options: either the completion deadline remains unaltered and the third reviewer’s input is disregarded altogether, or the deadline will be deferred to allow time for this reviewer’s comments to be taken into consideration. The new agreement is recorded in writing.*

5b) Are there any problems which prove impossible to be resolved at project level, whereby senior management might be of assistance?

*5b) If there are any problems, difficulties or obstacles which cannot be resolved by the project coordinator and/or the commissioner’s contact person, it is essential to refer the matter to a higher hierarchical level.*

*Example: In a project, the researchers are not provided with the data which has been promised by the commssioner, although the data is crucial to the project. The commissioner’s contact person says that he is unable to help. The researchers cannot proceed. The project coordinator then aligns with the head of department to discuss ways in which this problem can be resolved.*

5c) Has the senior management within both your institute and the commissioning organization been made aware of the (expected) research results and the product forms well in advance?

*5c) Senior management can enhance the effectiveness and assimilation of a product within the commissioning organization by drawing their counterpart’s attention to the forthcoming product at the earliest possible opportunity. Moreover, the discussion of relevant research results at the senior management level supports the profiling of your institute. It is important that senior management on both sides is aware of the forthcoming results and conclusions so that no one is taken by surprise. The proactive transfer of information about research results to senior management levels is therefore extremely worthwhile.*

*Example: Your institute is preparing a research report which has been commissioned by a specific policy directorate within the Ministry of Health Your director reported on progress and the expected results at a meeting of the Ministry’s Executive Council, whereupon a director at the Ministry realized that the report may well be relevant to his directorate and the Minister, which he then informed accordingly. The publication of the final report prompts an unexpected discussion between various field parties, a debate which inevitably attracts media attention. However, because your director already informed the Minister of Health about the publication and the contents of the report, the latter is able to field press questions when arriving at her next public engagement.*

*-----------*
