# Supplementary material for: Research for Policy (R4P): development of a reflection tool for researchers to improve knowledge utilization
Source: Implement Sci. 2016 Sep 30;11:133. doi: 10.1186/s13012-016-0496-1 (PMC5045649; doi:10.1186/s13012-016-0496-1)
Supplement: Supplementary file 3 — Final R4P tool. (PDF 358 kb) [file 13012_2016_496_MOESM3_ESM.pdf]

Research forPolicy

	Research Project		
	Formulation Phase	Production Phase	Extension Phase
**R4P TOOL**	**R4P questions**	**R4P questions**	**R4P questions**
Organizational environment of the project	**01<br>02<br>03<br>04<br>05<br>06**	**01<br>02<br>03<br>04<br>05<br>06**	**06**
Goal of the project	**07<br>08<br>09<br>11<br>13**	**07<br>09<br>10<br>11<br>12<br>13**	**13**
Interaction during of the project	**14<br>15<br>16<br>17<br>18**	**14<br>15<br>16<br>17<br>18**	**14<br>15<br>16<br>18**
Outcome of the project	**19<br>20<br>21<br>22<br>23**	**19<br>20<br>21<br>22<br>23**	**19<br>20<br>22<br>23**

I: Organizational environment of the project

1. What organizations will be involved in the project and in what capacity? What is the position of these organizations within the health domain of the project, and what are the practical implications of its position? Explanation and example 1

2. What are the (current) characteristics of the commissioning organization and its relationship with your organization? How do you address these factors during the process? Explanation and example 2

3. In what (dynamic) context does the commissioner operate during the project, and what implications could this have for the project itself? Explanation and example 3

4. In what dynamic context does your institute operate, and what implications could this have for your project and the alignment with the commissioner? Explanation and example 4

5. What are the specific characteristics of the researchers on your project team? How are tasks and responsibilities divided between them, and how is the work coordinated? Explanation and example 5

6. Are there any problems which prove impossible to be resolved at project level, whereby senior management might be of assistance? Explanation and example 6

II Goal of the project

7. How and when should the research product be used according to your organization and the commissioning organization? What is its purpose? Explanation and example 7

8. What is the underlying concern of the research question in relation to the problem that the commissioner wishes to solve? Explanation and example 8

9. To what extent is it possible to refine the research question at a later stage of the research process to bring it more closely in line with the commissioner's need? Explanation and example 9

10. Has the commissioning organization experienced any changes which may affect their knowledge need since the project proposal was produced? Does the research question formulated in the project proposal continue to address the current knowledge need? Is it necessary (and possible) to amend the project plan? Explanation and example 10

11. Within what timeframe does the commissioner require the product and why? Did you agree on the project schedule? Are all phases, milestones and deliverables clearly defined? Explanation and example 11

12. Have the scientific models, concepts and definitions to be applied within the project been agreed by all parties? Explanation and example 12

13. Did you explicitly agree on the rights and responsibilities of both the commissioner and your institute within the project? Explanation and example 13

III Interaction during the project

14. With whom should you align within your own organization during the process? Whose commitment must be sought and how? Explanation and example 14

15. With whom should you align externally during the process and whose commitment must be sought? Explanation and example 15

16. What type and frequency of consultation with the commissioner will be most appropriate to the process? Explanation and example 16

17. Did you plan any interim knowledge exchange with the commissioner regarding the content of the research product? Explanation and example 17

18. How do you achieve interim exchange of knowledge between the various users? Explanation and example 18

IV Outcome of the project

19. What types of user (at various levels within the commissioning organization) can be identified for the intended product, and how is their diversity to be taken into account in terms of content and process? Explanation and example 19

20. How do you monitor the production of (similar) knowledge products by other organizations, and the influence that such products may have on the commissioner and end users during the project? Explanation and example 20

21. Do you have a timely discussion with the commissioner with regard to the form of the research product? Explanation and example 21

22. Do you envisage to draft a plan for the presentation and dissemination of your research product, and has this been agreed well in advance with both the commissioner and relevant internal staff? Explanation and example 22

23. Has the senior management both your institute and the commissioning organization been made aware of the (expected) research results and the product forms well in advance? Explanation and example 23

Question 1

What organizations will be involved in the project and in what capacity? What is the position of these organizations within the health domain of the project, and what are the practical implications of its position?

Explanation question 1

It is important to identify: What are the characteristics of the different organizations in question? What is their position (in relation to your organization's network, e.g. commissioner, project partner, subcontractor). What authority or influence do they possess, and how can this be exploited in the interests of the project?

Example 1

The issue

An independent governmental organization is in charge of a database containing confidential health care information and this data is only provided for supervision purposes to the Health Care Inspectorate. Since your project is commissioned by the Health Care Inspectorate to support them in their tasks, the Health Care Inspectorate puts a data set from the database to your disposal. However, the independent governmental organization is not involved and raises objections to the use for research purposes in a meeting with your manager later in the process.

The outcome

To resolve the issue, your manager aligns with the organization and the Health Care Inspectorate. They agree to inform each other in future.

Question 2

What are the (current) characteristics of the commissioning organization and its relationship with your organization? How do you address these factors during the process?

Explanation question 2

It is important to identify: What are the characteristics of the commissioning organization (e.g. Health Care Inspectorate, Ministry of Health, or other ministry), including its institutional work culture. Attention should also be devoted to the commissioner's role within the research process (e.g. as a member of a steering group or advisory committee) and the degree of 'formal' involvement.

Example 2

The issue

Your commissioner, the Inspectorate, supervises health care practices in your country, maintains the law and as a result, is often the target of media attention. This clarifies why inspectors are expected to act both in an authoritative manner and in a very carefull way. In your own research project, the inspector involved wants to have full control and insists in being involved in decisions on methods and in publications. According to his workculture, he persists on his position despite your referring to your institute's independent position.

The outcome

Since this divergence of views can not be solved at project level, you ask your manager to align with the inspector's manager. They agree on the existing operating procedure ensuring both sufficient information for the Inspectorate and independence for the researchers. The inspector involved is informed by his manager on the agreed operating procedure and takes now another position in the project. In return, you commit to inform him on draft publications in order to avoid surprises.

Question 3

In what (dynamic) context does the commissioner operate during the project, and what implications could this have for the project itself?

Explanation question 3

To monitor the context in which the commissioner is operating, is essential to understand any unexpected course of action and to be able to discuss this with a view to understanding the commissioner's motives.

Example 3

The issue

At the commencement of your research project, the commissioner's contact person asks you to be kept fully informed of the decisions and choices made by your research team. Two months later, it turns out to be impossible to contact her in time, because she does not answer the phone, responds to emails only after a lengthy delay and, according to her secretary, has no time to schedule a meeting. Meanwhile, there have been media reports of an urgent reorganization within the commissioning organization, prompted by the minister's desire to show effective leadership following a number of incidents. This clarifies why the contact person is preoccupied with other matters.

The outcome

You align with the head of department to decide how best to proceed. You agree to delegate the issue to the account manager. She contacts the commissioning organization and via this route, your contact is restored.

Question 4

In what dynamic context does your institute operate, and what implications could this have for your project and the alignment with the commissioner?

Explanation question 4

External parties generally regard your research institute as a single, integrated organization. It is however important to devote attention to the activities and developments at the various levels within your institute and its individual departments in order to ensure uniformity, avoid overlap and address circumstances which may affect your institute as a whole. Matters to be considered include strategic plans, alignment between your institute and commissioning organizations at management level, new tasks and responsibilities for your organization, and other research projects or reports.

Example 4

The issue

Your institute publishes a report about the adverse effects of cycling in traffic, notably the inhalation of fine particulate matter from exhaust fumes. A week later, your institute publishes another report in which it advises local authorities to encourage people to cycle to work as a form of healthy exercise.

The outcome

The institute has unintentionally issued conflicting messages, causing confusion and attracting adverse media attention. The director of the institute is asked to explain this to both the Ministry of Health and the Ministry of Environmental protection. He commits to improve the institute’s internal communication.

Question 5

What are the specific characteristics of the researchers on your project team? Are tasks and responsibilities explicitly attributed to the team members? How is the work coordinated?

Explanation question 5

It is important to consider whether all necessary competences and expertise are represented within the team. The tasks should be clearly designated, for example to specialists in communications, media, design, data collection, etc. All team members should be aware of the purpose and deadlines of the product.

Example 5

The issue

In a major project, the project managers involve all relevant experts with respect to the complicated public health issues that will be investigated during the project. They carefully keep guard over the scientific quality of their reports and they present their findings at many acedemic occassions. Surprisingly, the commissioner expresses some dissatisfaction about the outcome. It turns out that this is caused by the many delays in the process and the unwelcome media message that came out of the public presentation of the final report.

The outcome

Apperently, necessary project management competences were not available at an adequate level. To avoid this type of difficulties in future, it was decided that to add a researcher with extensive process management competences to the project team and to put her in charge of managing the process.

Question 6
Are there any problems which prove impossible to be resolved at project level, whereby senior management might be of assistance?

Explanation question 6
If there are any problems, difficulties or obstacles which cannot be resolved by the project coordinator and/or the commissioner's contact person, it is essential to refer the matter to a higher hierarchical level.

Example 6
The issue
In a project, the researchers are not provided with the data which has been promised by the commissioner, although the data is crucial to the project. The commissioner's contact person says that he is unable to help since the data turn out to be confidential. The researchers cannot proceed.
The outcome
The project coordinator aligns with the head of department to discuss ways in which this problem can be resolved. In his turn, the head of department contacts the director, who discusses the issue with both his counterpart at the commissioning organization and they agree on the conditions for using the confidential data. After taking some measures, the researchers receive the data and continue with the project.

Question 7
How and when should the research product be used according to your organization and the commissioning organization? What is its purpose?

Explanation question 7
You need full clarity regarding the expectations which the product is to meet. In the case of a research report for the Ministry of Health, for example, the parties should agree the point within the policy process at which it is to be used, and the nature of that use.

Example 7
The issue
Your two-yearly reports are intended to provide input at different moments in the policy cycle. It is a major job to collect all data and to integrate them in a report every two years. However, policymakers indicate that policy realities constantly change and that they need more up-to-date data.
The outcome
After a constructive discussion, you agree to start a website to present all data. This website will be updated four times a year.

Question 8
What is the underlying concern of the research question in relation to the problem that the commissioner wishes to solve?

Explanation question 8
You need clarity with regard to whether the research question and its interpretation adequately address the commissioner's need. Conversely, and depending on the type of product and the anticipated results, it may also be necessary to examine whether the commissioner will be able to implement an adequate response based on the research findings and whether adequate policy instruments are available to do so. You have to be aware that the commissioner can be confronted with queries or criticism on the research from the professional field, parliament, the media, etc.

Example 8
The issue
The Ministry of Health submits a research question to investigate prevention of obesity in children. The question is rather vaguely articulated and for the researchers, it is not clear what exactly made the commissioner submit the question. Is there need for information on the trends in the number of obese children, on their geographical distribution, on effectiveness of interventions, on effectiveness of public campaigns, on the role of youth health care? All these issues have been addressed already. In a discussion with policymakers, they indicate that obesity in children will become a key issue in a new national prevention plan starting soon. The research should explore (policy) options to enhance healthy food uptake and physical exercise at a local level. The results are awaited within half a year.
The outcome
It is agreed to discuss any findings after three months to align on recommendations. The results will be presented in an infographic that can be used by local policymakers.

Question 9

To what extent is it possible to refine the research question at a later stage of the research process to bring it more closely in line with the commissioner's need?

Explanation question 9

A 'broad' research question is not necessarily a bad thing since you will have greater discretion to define your own focus, which will emphasize the organization's independent scientific position. You don't have to specify all research requirements in advance if you create opportunities for further refinement and coordination during the research process (e.g. by means of scheduled consultation meetings).

Example 9 a)

The issue

The Ministry of Health requests a Public Health Status and Forecasts report for which the research question is very generally formulated. It is agreed that defining the themes is an integral part of the project.

The outcome

In the first period of the production phase, the researchers can take certain time to reflect on the themes and to align with policymakers and scientists in planned meetings. They finish the report in time.

Example 9 b)

The issue

The Inspectorate asks for a model to select inspection objects. At the start of the project, the researchers need a lot of time to find out what the inspectors exactly expect and need.
This extension of the formulation phase was not foreseen in the project plan and causes delay already at the start of the project. At a later stage, lack of both time and budget hinder the project.

The outcome

For future projects, the project manager firmly resolves to plan time and budget for further definition of the project in the production phase to avoid problems at the final stage.

Question 10

Has the commissioning organization experienced any changes which may affect their knowledge need since the project proposal was produced? Does the research question formulated in the project proposal continue to address the current knowledge need? Is it necessary (and possible) to amend the project plan?

Explanation question 10

The commissioner's requirements with regard to the purpose or form of the end product may change over time, even after approval of the original project proposal. It is important to ascertain whether this is the case on a regular basis, and to consider whether any changes to the process itself are then necessary (and possible).

Example 10

The issue

A commissioner wishes to commission research examining the risks of a new technology used in hospitals, as a follow-up to an earlier study. You formulate a proposal but before the project itself starts, a serious incident involving a different type of technology takes place. This causes significant media attention and the commissioner is subject to severe criticism. It is now far more important for the commissioner to gain further knowledge about this new technology than about that covered by your original proposal.

The outcome

In consultation with you, a new focus and a different approach are adopted.

Question 11

Within what timeframe does the commissioner require the product and why? Did you agree on the project schedule? Are all phases, milestones and deliverables clearly defined?

Explanation question 11

The exact moment of the product’s finilization and/or presentation can be important in terms of its relevance and use.

Example 11

The issue

The commissioning Ministry requires an extensive research report to support an important policy document which is subject to a very strict deadline. Given the deadline not all required research can be performed within time, but finilization after the deadline will make the product far less relevant to the commissioner.

The outcome

The project team agrees on the minimum part of the product to be delivered before the deadline.

Question 12

Have the scientific models, concepts and definitions to be applied within the project been agreed by all parties?

Explanation question 12

The scientific models, concepts and definitions used during the research can have a significant bearing on the findings. It is possible to dismiss an unfavourable research result by arguing that the approach was flawed. To ensure the acceptance and relevance of the product, it is prudent to create support for the scientific basis among all stakeholders, including the commissioning organization. It is also important to identify any (political) sensitivities about the models or definitions.

Example 12

The issue

In your project, you have to calculate the cost of a certain type of medical care. You find that this type of medical care has various definitions in practice, with different interventions and equipment being grouped under the same general heading. For the purposes of your research, you opt to apply the definition followed by the majority of health care professionals and for which reliable data sources are available.

The outcome

When the draft report is completed, the high cost calculation takes the commissioner unpleasantly by surprise. The commissioner argues that your organization did not apply the correct definition. It takes a long discussion to convince the policymakers of the correct method. To compensate for the commissioner’s objection, you decide to extend the discussion on the definition used in your report.

Question 13
Did you explicitly agree on the rights and responsibilities of both the commissioner and your institute within the project?

Explanation question 13
General rules establish the rights and responsibilities of the parties involved in a project of your institute. However, it is also important to examine whether any specific agreements are required with regard to aspects such as the ownership of data, the procedures for providing feedback on draft versions of the product, and intellectual property rights.

Example13
The issue
A commissioning organization wishes to have three of its staff review a draft report. However, one of the three is extremely slow to do so. Clear agreements have been made with regard to the review process and the action to be taken further to the reviewers’ comments (Your institute retains full editorial control and will decide whether to make any suggested changes). In addition a time limit has been set, i.e. the annotated version must be returned within two working weeks.
The outcome
The research coordinator decides to present the commissioner’s contact person with two options: either the completion deadline remains unaltered and the third reviewer’s input is disregarded altogether, or the deadline will be deferred to allow time for this reviewer’s comments to be taken into consideration. The new agreement is recorded in writing.

Question 14

With whom should you align within your own organization during the process? Whose commitment must be sought and how?

Explanation question 14

Vertical imbedding of the product within your organization is important for its legitimacy towards the commissioner, the resources and capacity required (determined in part by the desired quality and form of the product) and own future use or further research.

Example 14

The issue

During the production phase of a major project, the project team informs the director on the proceedings in regular meetings. In the final stage of a theme report, a director at the Ministry of Health is dissatisfied with its publication form, a flyer instead of a formal report and he immediately contacts the director of the research institute.

The outcome

Being well informed, the director of the research institute is able to respond adequately on the complaint. The directors agree on a compromise, a formalized brochure and additional budget to complete a research report.

Question 15

With whom should you align externally during the process and whose commitment must be sought?

Explanation question 15

In advance of the research process, you should identify the persons with whom alignment is required (also with a view to the intended recipient of the product). You should also determine how contact can best be established and maintained.

Example 15

The issue

Your institute is asked to conduct a major research project on public health. Two other research institutes are very disappointed that they were not charged with this project.

The outcome

Since you need their expertise and wish to maintain a good relationship, you decide to invite them for a stakeholder meeting before the project starts. You agree to include several experts from both institutes in the project team and to organize a management meeting twice a year. All organizations are satisfied with this outcome.

Question 16

What type and frequency of consultation with the commissioner will be most appropriate to the process?

Explanation question 16

You should ensure that both capacity and the time schedule allow for adequate interaction and consultation. Agree the form that such consultation will take (e.g. face-to-face, via social media, email etc.) with all parties concerned.

Example 16

The issue

The contact persons of the commissioning organization are very busy people and it is difficult to contact them. At the start of the project, you agree on different communication forms. Four meetings are planned for the whole year. Furthermore, you start an online community to discuss upcoming issues in the mean time. Any interim questions will be solved by e-mail or by phone.

The outcome

After several months, it turns out that the online community is hardly used. However, the planned meetings prove to be very important for alignment with the commissioner. In the next meeting with the commissioner, you agree to plan an extra meeting and to change the function of the online community into a repository of background information.

Question 17
Did you plan any interim knowledge exchange with the commissioner regarding the content of the research product?

Explanation question 17
The intended effect of the research product can be increased or achieved somewhat sooner by scheduling discussions of the interim results (before completion of the project and its final report). It is important to discuss possible research outcomes with the commissioner at the earliest possible opportunity, in order to allow all parties to be adequately prepared for the implications of a particular finding.

Example 17
The issue
You foresee that your research results will not be politically expedient for the Ministry of Health. In an early stage, you share the results with your contact person and with your manager.
The outcome
Although the Ministry is not in the positin to influence your research results, timely discussion with policymakers give them the opportunity to prepare an adequate response before your report is published. It must be remembered that such discussion relates solely to alignment and anticipation. It does not entail the commissioner's 'approval' of the results. Your institute maintains its scientific independence and integrity by ensuring appropriate internal alignment between all hierarchical levels of the organization.

Question 18

How do you achieve interim exchange of knowledge between the various users?

Explanation question 18

The impact of the research product will be enhanced when there is interim discussion and an exchange of knowledge between the various end users during the course of the project. It is therefore useful for the project coordinator to know who alignst with those end users on behalf of the commissioner, and what activities he or she undertakes in order to do so. If the commisioner's alignment efforts are not enough to ensure knowledge exchange with the end users, it may be necessary for the project team to take action, involving the account manager and his/her staff.

Example 18

The issue

A project has a steering group on which various directorates of the Ministry of Health are represented. The project group collates and processes the steering group's comments on the draft versions of the final research report. Some directorates offer considerable feedback, others little or none at all.

The outcome

After publication of your report, the project evaluation reveals that the directorates which have provided least feedback also show least awareness of the report's findings, yet are the most critical with regard to its practical value. A steering group member who shows considerable involvement and engagement in the process tends to engender greater satisfaction. The project team decides that any follow-up project will involve closer alignment with the commissioner's contact person in order to determine the exact membership of the steering group and the degree of involvement its members are expected to show.

Question 19

What types of user (at various levels within the commissioning organization) can be identified for the intended product, and how is their diversity to be taken into account in terms of content and process?

Explanation question 19

There may well be several types of user within the commissioning organization (e.g. different policy directorates, departments or hierarchical levels). Their diverse interests may demand differentiation in terms of the presentation of the product, both in form and timing.

Example 19

The issue

For your project, you regard the policymakers of the Ministry of Health as the intended users of the knowledge products resulting from your project. All directorates are represented in the advisory board of the project and you expect that your knowledge products will be widely supported within the Ministry.

The outcome

In practice, it turns out that behind the scenes, your first report is not supported unanimously within the Ministry and some policymakers question whether your research should be continued. You find that this mismatch can be attributed to the characteristics of the policy directorates: one directorate acts more on general or system level whereas another is more specialized in a specific health domain. This causes some directorates to feel less committed than others, although during the advisory board meetings, the representativesdo not clearly express their discontent. You decide to spend frequently a work day at the Ministry to enable informal meetings with representatives of the different directorates and to gather information on their specific needs.

Question 20

How do you monitor the production of (similar) knowledge products by other organizations, and the influence that such products may have on the commissioner and end users during the project?

Explanation question 20

Throughout the production phase, it will be useful to ascertain whether other research organizations are working on similar or related products. It is also important to determine the likely value of such products to the commissioner, and whether they will affect the (perceived) relevance of your product. This will enable you to take these influences into account in your project and in the alignment with the commissioner.

Example 20

The issue

Another research organization intends to present a report at a large event just days before you are due to publish a product relating to similar subject matter. This will inevitably distract attention from your product. Moreover, inside information suggests that the conclusions of the other report directly contradict your own findings. This could place the commissioner in a particularly difficult situation.

The outcome

You decide to contact both the commissioner and the higher hierarchical levels within your institute to align on the potential problems and the action required.

Question 21

Do you have a timely discussion with the commissioner with regard to the form of the research product?

Explanation question 21

Much research culminates in the publication of a report. However, it is possible that a different form of product will be more useful to the commissioner. Examples of alternative products include a summary of a scientific publication to support a policy decision, a presentation, or an instrument such as a simulation model or a survey questionnaire. Knowledge presented during a round-table discussion is also a ‘product’.

Example 21

The issue

A commissioner faces a particularly complex policy issue and requests your institute to provide input for its internal deliberations. Out of habit, your commissioner asks for an official report on the subject. However, you realize that a report will not meet the commissioner’s knowledge need for their own exploration of the problem and can not be delivered on time.

The outcome

Following consultation with the commissioner, it is agreed to prepare and host a brainstorming session for the policy-makers concerned. Based on literature study, your team will also produce a background document and a presentation. On the outcomes of the brainstorming session, the commssioner is able to base further action and decisions, and also to formulate terms of reference for a research project on the same topic to be conducted the following year.

Question 22

Do you envisage to draft a plan for the presentation and dissemination of your research product, and has this been agreed well in advance with both the commissioner and relevant internal staff?

Explanation question 22

Working to a set plan for the presentation and dissemination of the research product will increase its outreach and hence the likelihood that the findings are acted upon in practice. It is useful to plan the relevant activities during the course of the project itself, since there may be neither time nor capacity once the product has been completed

Example 22

The issue

Despite some delays in a project, the deadline can still be met if staff works overtime. The research product is completed just before the Christmas holiday, much to the relief of the project team. When work resumes in January, the team is disbanded and its members assigned to new projects. They have no time left for extended presentation or dissemination of the former project's results since these activities had not been planned and no budget is available.

The outcome

During the new commissioning cycle by the Ministry, the researchers realize that their previous research efforts did little to raise awareness of their findings. The policymakers show scant interest in the research findings and the Ministry commissions only limited further research. The researchers discuss this disappointing result in their team and agree to plan more time and capacity for dissemination in a new project they are working on.

Question 23

Has the senior management within both your institute and the commissioning organization been made aware of the (expected) research results and the product forms well in advance?

Explanation question 23

The senior management can enhance the effectiveness and assimilation of a product within the commissioning organization by drawing their counterpart's attention to the forthcoming product at the earliest possible opportunity. Moreover, the discussion of relevant results at the senior management level supports the profiling of your institute. It is important that the higher organizational levels on both sides are aware of the forthcoming results and conclusions so that no one is taken by surprise. The proactive transfer of information about research results to the higher management levels is therefore extremely worthwhile.

Example 23

The issue

Your institute is preparing a research report which has been commissioned by a specific policy directorate within the Ministry of Health Your director reports on progress and the expected results at a meeting of the Ministry's Executive Council, whereupon a director at the Ministry realizes that the report may well be relevant to his directorate and the Minister, which he then informs accordingly.

The outcome

The publication of the final report prompts an unexpected discussion between various field parties, a debate which inevitably attracts media attention. However, because your director already informed the Minister of Health about the publication and the contents of the report, the latter is able to field press questions when arriving at her next public engagement.
